# The Army Medical Service

**Published:** 1901-05-11

**Authors:** 


### The Hospital

A Journal of

The Medical Sciences and Hospital Administration.

Vol. XXX.—No. 763] Edited by Sir Henry Burdett, K.C.B., and Solomon C. Smith, M.D., M.R.C.P. [May 11, 1901.

### The Army Medical Service.

That profoundly uninteresting body, the Parliamentary Bills Committee of the British Medical Association, has once more been undergoing the pangs of parturition, and has been delivered of a feeble and misshapen infant, which the utmost care of the Council of the same Association will certainly be unable to rear. The infant in question purports to be neither more nor less than a final deliverance of the mind of the Association (*sit venia verbo*) upon the question of the proper organisation of the medical service of the Army The lengthy document which has been prepared by a sub-committee, adopted by a complete committee, and sanctioned by the Council, stands as a monument of the incapacity of its authors to distinguish between the essential and the trivial, or between the principles and the accidents by which the relations of the medical profession to the Army are for the moment controlled. Anything more ludicrous than the application said to have been made by the sub-committee to the authorities of various seats of learning, enumerating eighteen heads of possible dissatisfaction with the service on the part of Army surgeons, and requesting the authorities in question to classify these heads in the order of their relative importance, has seldom fallen under our notice; and it is eminently creditable to the sagacity of the authorities of the Universities of Oxford, Cambridge, and London, who could hardly all have been overlooked even by a sub-committee of the British Medical Association, that no replies from them appear to have been received. The whole case may be put absolutely in a nutshell. Army surgeons are underpaid, they are overworked, and the conditions of the service are rendered artificially unpleasant by the line of demarcation which separates them from the so-called "combatant" officers. The only possible counterpoise to these disadvantages, and a counterpoise which would be effective in precise proportion to the elevation of mind of the surgeon and to his devotion to his duties would be furnished by large opportunities of doing good professional work, and by reasonable security that the character of such work would be appreciated when it was done. This counterpoise is absolutely wanting. The qualities which bring a physician or surgeon to the head of his profession in civil life would have no tendency to promote his advancement in the Army, in which no higher merit is recognised than self-effacement beneath routine. It follows that men who are conscious of the possession of powers which would be likely to lead to success in practice do not, as a rule, care to bury these powers in a service in which they would be wholly wasted. The case bears some analogy to that of the medical care of the insane. It is believed by many that the slenderness of the progress which has been made in the pathology and treatment of insanity, as compared with those of other forms of disease of the nervous system, is largely due to the extent to which the attention of the medical superintendents of asylums is withdrawn from strictly professional work by the claims made upon their time and thoughts as the governors of great establishments. One of the most powerful of Moritz Retsch's etchings, "The Fate of the Poet," represents a follower of the Muses who had descended from his Pegasus to bestride a sorry steed of earthly lineage, and who, in riding across a ford, is dragged from his seat and drowned by the water nymphs. The fate of the medical profession in the Army is something not dissimilar. The "combatant" officer cherishes an uneasy sense that the doctor (if he be a good doctor) is intellectually his superior, and tries to "take it out of him" in some other way. The authorities at the War Office do not in the least understand the work which the doctor is called upon to do, and only seldom appreciate its importance, or recognise the conditions under which alone it can be satisfactorily accomplished. A recent example proves that, if they are so fortunate as to get hold of a man of exceptional abilities, they do not know either how or where to employ those abilities for the public benefit. We doubt whether it would be reasonable to hope that they should. It is not beyond the capacity of the War Office to understand that if they want good men they must pay the market price for them, but more than this is hardly to be expected. An important step towards securing a really effective military medical service would be the association with the purely military authorities, whether combatant or medical, of physicians or surgeons of eminence in civil life as advisory colleagues. If all important questions bearing upon the work of the department were reported on by a committee, two members of which were appointed on the nominations of the College of Physicians and of the College of Surgeons, the hopeless blundering of the past would be rendered, if not impossible, at least inexcusable, for the future.

